# Characterization and Screening of Thermophilic *Bacillus* Strains for Developing Plant Growth Promoting Consortium From Hot Spring of Leh and Ladakh Region of India

**DOI:** 10.3389/fmicb.2018.01293

**Published:** 2018-06-26

**Authors:** Jay Prakash Verma, Durgesh Kumar Jaiswal, Ram Krishna, Satya Prakash, Janardan Yadav, Vijai Singh

**Affiliations:** ^1^Institute of Environment and Sustainable Development, Banaras Hindu University, Varanasi, India; ^2^Hawkesbury Institute for the Environment, Western Sydney University, Sydney, NSW, Australia; ^3^Department of Soil Science and Agricultural Chemistry, Institute of Agricultural Sciences, Banaras Hindu University, Varanasi, India; ^4^Synthetic Biology Laboratory, Department of Microbiology, School of Biological Sciences and Biotechnology, Institute of Advanced Research, Gandhinagar, India

**Keywords:** thermophile, extremophile, hot spring, *Bacillus*, microbial consortium, genetic diversity, ISSR, plant growth attributes

## Abstract

In the present investigation, the main aim is to identify and characterize the potential drought tolerant plant growth promoting consortium for agricultural productivity. Three bacterial isolates were isolated from hot spring of Chumathang area of Leh district. *Bacillus* species (BHUJP-H1, BHUJP-H2, and BHUJP-H3) were done some biochemical tests including catalase, cellulase, amylase, indole-3-acetic acid, phosphate solubilisation, production of ammonia, siderophore, and hydrogen cyanide. Molecular characterization of isolates was done by 16S rDNA sequencing, e.g., *Bacillus subtilis* BHUJP-H1 (KU312403), *Bacillus* sp. BHUJP-H2 (KU312404) and *B. licheniformis* BHUJP-H3 (KU312405). The genetic diversity of the isolates was assessed by seven inter simple sequence repeat, all primer shows high polymorphism. The highest polymorphism efficiency and polymorphism information content showed by UBC-809 and UBC-836 which were 100% and 0.44 respectively, the lowest is by UBC-807 75% and 0.28 respectively. On an average 90.69% polymorphism efficiency and 0.40 polymorphism information contents obtained by used markers. The highest, 11.08 and the lowest, 4.50 effective multiplex ratios obtained for primer UBC-823 and UBC-807, on an average 7.99 effective multiplex ratio obtained. The highest, 4.89 and the lowest, 1.25 marker indexes obtained by UBC-836 and UBC-807 respectively and on an average 3.24 obtained. The UPGMA cluster analysis divided a population into two clusters I and II, in which BHUJP-H1 and BHUJP-H2 grouped under same while BHUJP-H3 grouped under another cluster. The treatment combination of *Bacillus subtilis* BHUJP-H1, *B. subtilis* BHUJP-H1+ *B. licheniformis* BHUJP-H3 and *B. subtilis* BHUJP-H1+ *Bacillus* sp. BHUJP-H2+ *B. licheniformis* BHUJP-H3 were recorded better combination for enhancing plant growth attributes of *Vigna radiata* as compared to control and others. The plant growth promoting consortium, e.g., *Bacillus subtilis* BHUJP-H1, *Bacillus subtilis* BHUJP-H1+ *B. licheniformis* BHUJP-H3 and *B. subtilis* BHUJP-H1+ *Bacillus* sp. BHUJP-H2+ *B. licheniformis* BHUJP-H3 can be further used as effective microbial inoculant for enhancing the production of mungbean in field conditions. *Bacillus* sp. BHUJP-H1 and *Bacillus* sp. BHUJP-H2 may use as drought tolerant plant growth promoting consortium for enhancing the sustainable agricultural productivity.

## Introduction

Chumathang area of Ladakh region of Indian subcontinent forms a part of the tectonically active belt having many hot springs (Siangbood and Ramanujam, 2011). In general, hot springs are of utmost importance because of the unique characteristic thermophilic biocatalysts produce by thermophilic microorganisms thriving under the extreme temperature condition. Thermostable enzymes such as protease, lipase, amylase, cellulase, phosphatase, asperginase, esters, and carboxylase from thermophiles are gaining more attention due to the medical, industrial and agriculture benefits. Lele and Deshmukh (2016) and Mohammad et al. (2017) reported that the various *Bacillus* spp. has been isolated from hot springs which has ability to produce thermostable enzymes. Therefore, the hot springs are recently gained more interest toward the production and enhancement of enzymes, sugars, compatible solutes and antibiotics (Satyanarayana et al., 2005). However, biodiversity of such extreme environments, the microbes have received several attentions because of their diverse and unique ecology, chemistry and the opportunity to identify a novel molecule (Kuddus and Ramtekke, 2012). Although, the possibility of the presence of new microbes with high economic and industrial values, there are limited reports available on the microbial diversity of hot springs from India (Panda et al., 2015). As the culture dependent studies are valuable for isolating microorganisms and exploring their properties, the culture independent methods offer a more comprehensive assessment of microbial diversity (Tringe and Hugenholtz, 2008). *Bacillus licheniformis* was isolated and molecularly characterized by Saharan and Verma (2014) which show the ability of multiple plant growth promoting characteristics such as ammonia production, indole acetic acid production, phosphate solubilization, catalase production, heavy metal tolerance and ACC deaminase activity. isolate is a potential PGPR candidate for enhancing sustainable agriculture. Gutiérrez-Manero et al. (2001) reported that the *B. pumilus* and *B. licheniformis* have been documented to produce gibberellins. Other scientist reported that *B. licheniformis, B. cereus, B. circulans, B. subtilis*, and *B. thuringiensis* found as potential biocontrol agents having chitinolytic activities (Sadfi et al., 2001). The strains *Bacillus licheniformis* SB3086 secreted the Novozymes by their spores which play an important role as phosphate solubilizer strain and it is also effective against Dollar spot disease of plants (Saharan and Verma, 2014). Other scientist Kayasth et al. (2013) reported that the *Bacillus licheniformis* identified and explored as the potential PGPR strain to be developed as multifunctional biofertilizer for multiple crop production.

Thermophilic microorganisms have been recognized for its abundant significance in the industry owing their capability to function at the extreme environmental condition. The microbial diversity in various hot springs from United States, Russia, Iceland, Algeria, New Zealand, and India have recently been investigated and identified by using 16S rRNA sequence from culture-independent or culture-dependent methods (Claus and Fritz, 1989; Reysenbach et al., 1994, 2000; Huber et al., 1998; Ghosh et al., 2003; Van den Burg, 2003; Nazina et al., 2004; Schaffer et al., 2004; Belkova et al., 2007; Kecha et al., 2007; Ghati et al., 2013). Hot spring metagenomics suggests that the dominant phylum and the dominant taxa within each phylum in distinct hot spring which depends on temperature, pH, and geochemistry of waters. The need of more depth analysis requires understanding the total bacterial diversity of hot springs. In a recent study, the *Bacillus* strains have been isolated and tested by biochemical and molecular levels. These strains were recovered from the wide range of extreme environments of Atri and Taptapani hot springs of Odisha (Cihan et al., 2012). In India, the hot spring of Bakreshwar, Balarampur, Chumathang, Panamic, Manikaran, and Vashisht have been characterized as rare places which can be potential sources of novel genes and microorganisms with a unique characteristic (Sharma et al., 2009; Kumar et al., 2013).

ISSR-PCR tools and techniques is very good and effective for genetic diversity analysis. ISSR (inter simple sequence repeat) is a quick and cheap molecular marker techniques for multiple application regarding the categorization of genetic similarity among populations and species (Baysal et al., 2011). The complementary sequences between two neighboring microsatellite is applied as PCR primers; the variable region among them converts amplified. The choice of ISSR was depended on their comparative practical easiness, level of polymorphisms, cheaper technology, simply relevant for prokaryote and eukaryote for amplifying which sequences that are more copious during evolution (Kumar et al., 2013).

There are recent advancement of tools and technologies, the extremophilic microorganisms identified as a potential sources of novel pigments (as food additives), enzymes like cellulases which can be valuable in agriculture as inoculants (plant growth-promoting bacteria) or bio control agents in extreme habitats (Khan and Patel, 2007; Srinivas et al., 2009). Another importance of extremophilic strains application can be used as the enhancement of soil productivity and fertility where the soil pH is extreme, it may be more saline or acidic. In this circumstances, extremophiles microbial consortium will be better and novel aspect for sustainable agricultural production. Therefore, the stress resistant microbial diversity of Bacillaceae and Paenibacillaceae can be identified and characterized in these extreme environments. In acidic soils, the availability of essential nutrients like, phosphorus, calcium, magnesium, and molybdenum are affected. Some studies are reported on the dissemination and multiplicity of bacteria in acidic soils (Perez et al., 2007; Yadav et al., 2011; Verma et al., 2013). The arid deserts have different types of microbial communities that can persist extreme environment including hot temperature and low moisture. Such environments encompass typically poor soils quality with low organic content and limited amounts of bioavailable inorganic nutrients. The microbiota of desert ecosystems is not only responsible for enhancing the productivity, biogeochemical cycling of elements and ecosystem balance, but also for soil neogenesis and improvement of soil structure. The balance of soil carbon storage is depended upon microbial activities in response to the climate change which will partially control and loss under future temperature and precipitation conditions. The aim of present study is to isolate and characterize bacterial species to develop plant growth promoting consortium for enhancing sustainable agriculture.

## Materials and Methods

### Isolation of Microorganisms

The soil and water samples were collected from hot spring of Chumathang of Leh district, Ladakh region of Jammu and Kashmir, India. Isolation of microorganisms was done by serial dilution and plating methods. We took 1 g of moist soil in a test tube that contains 9 ml of sterile saline water (0.85% NaCl) and mixed properly then serially diluted up to 10–7. Total 100 μl aliquots of each dilution were transferred and spread aseptically on different agar plates such as Nutrient agar, Kenknight and Munaiers agar, Potato Dextrose Agar, Tryptone Soya agar, Pikovskaya agar, and King’s B Base. The plates were incubated for 2–5 days at 45°C. After incubation, the different microbial colonies were found on plates. We counted and calculated the total numbers of colonies forming unit (CFU). Subsequently, the different types of colonies were picked up and streaked on respective plates for further purifying a single and pure colony. The presumptive isolates of phosphate solubilizers were screened and selected on the basis of halo zone produced in Pikovskaya agar. All microbial isolate was sub-cultured on their respective medium by the streaking method to get pure colonies and stored on slant media at 4°C and also glycerol stock in -80°C for further use. We have isolated three microbes which showed more effective and fast growth on high temperatures like 45, 50, and 60°C.

### Morphological and Biochemical Properties of Microbes

Microorganisms were characterized according to morphological characteristics such as bacterial isolates colony margin, shape and color (Kloepper et al., 1992; Gilbert and Jack, 1993) and biochemical assays including, Gram staining, amylase, catalase and cellulase test (Cappuccino and Sherman, 1992; Aneja, 2003) (**Tables 1**, **2**).

**Table 1:** Morphological characters of isolated bacterial strains from hot spring.

Strains	Cell morphology		Colony morphology			
	Gram staining	Shape	Form	Elevation	Margin	Color
*B. subtilis* BHUJP-H1	Positive	Rod	Circular	Flat	Curried	Creamy white
*Bacillus* sp. BHUJP-H2	Positive	Rod	Spindle	Flat	Undulate	Whitish
*B. licheniformis* BHU-H3	Positive	Rod	Filamentous	Flat	Curried	Whitish

**Table 2:** Biochemical characterization of isolated bacterial strains.

Strains	Biochemical characterization			Growth at different temperature				
	Amylase	Catalase	Cellulase	30°C	40°C	50°C	60°C	80°C
*B. subtilis* BHUJP-H1	+	++	++	+	+	+	++	-
*Bacillus* sp. BHUJP-H2	-	++	+	+	+	+	+	-
*B. licheniformis* BHU-H3	+	++	++	+	+	+	+	-

*Single positive sign (+): normal activity showed; double positive sign (++): more activity showed of particular enzyme and growth by bacterial strains; negative sign (-): no enzyme activity and no growth*.

### Plant Growth Promoting Properties of Thermophilic *Bacillus* Strains

Plant growth promoting properties such as Indole-3-acetic acid (IAA) was estimated in unit μg/ml of broth culture and performed by methods of Bric et al. (1991) (**Table 3**). The phosphate solubilization activity was estimated in unit μg/ml of broth culture and performed on Pikovskaya agar medium containing tricalcium phosphate (Pikovskaya, 1948) followed by Ammonium bicarborate diethylene triamine penta acetic acid (AB-DTPA) method (Soltanpour and Workman, 1981) and soluble phosphorus was determined by Ascorbic acid method (Watanabe and Olsen, 1965) (**Table 4**). HCN production test was done by adapting the method of Lorck (1948). Additionally, the ammonia production was evaluated by the methods of Cappuccino and Sherman (1992).

**Table 3:** Estimation of IAA production in thermophilic *Bacillus* strains in broth cultures at different concentration of tryptophan concentrations.

Bacterial strains	IAA production (μg/ml) at different incubation time					
	150 μg/ml tryptophan concentration			300 μg/ml tryptophan concentration		
	24 h	48 h	72 h	24 h	48 h	72 h
*B. subtilis* BHUJP-H1	14.65 ± 0.66^a^	17.12 ± 0.13^a^	18.35 ± 0.33^a^	21.36 ± 1.29^a^	19.08 ± 1.15^a^	24.85 ± 0.36^b^
*Bacillus* sp. BHUJP-H2	25.41 ± 0.52^c^	26.36 ± 1.15^c^	24.06 ± 0.12^b^	25.74 ± 1.31^b^	22.94 ± 1.91^b^	22.27 ± 0.30^a^
*B. licheniformis* BHUJP-H3	20.93 ± 1.26^b^	24.34 ± 0.63^b^	31.79 ± 1.57^c^	24.55 ± 0.88^b^	32.52 ± 0.63^c^	34.76 ± 1.12^c^

*IAA, Indole-3-acetic acid (μg/ml) at 150 and 300 μg/ml tryptophan as precursor; ^*a*^Values are the mean ± SD (standard deviation), Mean values in each column with the same superscript (s) do not differ significantly but different superscript is showed significantly different between each treatments by Duncan *post hoc* multiple comparison tests (*P* ≤ 0.05)*.

**Table 4:** PGPR activities of isolated thermophilic *Bacillus* species.

Bacterial strains	Phosphate solubilisation (μg/ml)		NH_3_	HCN	Siderphore
	3 days	6 days			
*B. subtilis* BHUJP-H1	12.09 ± 0.89^a^	22.48 ± 1.47^a^	+	++	+
*Bacillus* sp. BHUJP-H2	12.76 ± 1.15^a^	63.14 ± 0.97^c^	+	+	+
*B. licheniformis* BHU-H3	33.01 ± 0.91^b^	42.14 ± 1.45^b^	+	-	-

^a^*Values are the mean ± SD (standard deviation), Mean values in each column with the same superscript (s) do not differ significantly but different superscript is showed significantly different between each treatment by Duncan *post hoc* multiple comparison tests (*P* ≤ 0.05); Single positive sign (+): normal activity showed; double positive sign (++): more activity showed of particular properties like HCN (hydrogen cyanide) by bacterial strains; negative sign (-): no activity*.

### Effect of Organophosphate Insecticide on Growth of *Bacillus* Strains and Their Interaction

Disk diffusion method was used for growth of microbes with insecticide. Tolerance levels of microbial strains with different concentration of insecticide were determined using the filter paper disk technique. These techniques also used for test of antibiotics resistance of microbes (Bauer et al., 1966) and later used to test the effect of insecticide on microbial growth (Mallik and Tesfai, 1983; Martensson, 1992). Monocrotophos insecticide commercial name Monocrown 36% SL was obtained from the market. The recommended dose of Monocrotophos is 0.8 mL/L (0.8 μL/mL) of water. The recommended dose of Monocrotophos was diluted to 1X (0.8 μL/mL), 2X (1.6 μL/mL) and 10X (80 μL/mL) using the same solvent (water). Others, chlorpyrifos insecticide commercial name Messban 20% EC was obtained from the market. The recommended dose of chlorpyrifos is 2 mL/L of water. The recommended dose of chlorpyrifos was diluted to 1X (2 μL/mL), 2X (4 μL/mL), 3X (6 μL/mL), and 10X (20 μL/mL) using the same solvent (water). Sterile filter paper disk was used for insecticide test. Sterile filter paper disk was used for insecticide test. The sterilized disks were dipped in different concentration of insecticides and put on respective microbial inoculated plate of nutrient agar under laminar air flow. Control disk was dipped with sterile distilled water and put on respective media with inoculated strains. Insecticide disks were put on the nutrient agar plate which is uniformly spread with a pure culture of different microbial strains. The plates were then incubated at 30°C for 48 h. Thus, each plate contained four disks of different concentrations of monocrotophos insecticide (Control, 1X, 2X, and 10X) were prepared for the experiment. While, four disks of Control, 1X, 2X, 3X, and 10X concentrations of chlorpyrifos was prepared for the experiment. After 48 h plates were observed for the zone of inhibition around the disks (**Table 5**).

**Table 5:** Inhibition zone of monocrotophos (monocrown 36% SL) and chlorpyrifos (Messban 20% EC) on growth of microbes6.

Strain	Monocrotophos after 48 h					Chlorpyrifos after 48 h				
	1x	2x	3x	10x	Response	1x	2x	3x	10x	Response
BHUJP-H1	-	-	-	-	Tolerant	+	+	+	+	Susceptible
BHUJP-H2	-	-	-	-	Tolerant	+	+	+	-	Susceptible
BHUJP-H3	-	-	-	-	Tolerant	-	-	-	-	Tolerant

*Single positive sign (+): showed growth inhibition zone against insecticide concentration; negative sign (-): no inhibition zone that means bacteria grow on media plate against insecticide concentration; 1x, 2x, 3x, and 10x mean insecticide concentration increasing with respect of recommended doge in agricultural field for pest control*.

### Molecular Identification of Isolated Microbial Strains

#### Genomic DNA Extraction From Microorganisms

The single colony was grown in nutrient broth at 28 ± 2°C in shaker incubator with 120 rpm for overnight. Genomic DNA extraction was isolated by using methods described by Sambrook and Russel (2001). Genomic DNA was checked on a 0.8% (w/v) agarose gel electrophoresis containing ethidium bromide and it was run with 100 V for 45 min in 1X TAE buffer (0.04 M Tris acetate, 0.001 M EDTA) along with EcoR1/Hind III double digest Lamda DNA marker (Banglore Genei, Pvt., Ltd., Bangalore, India).

#### Amplification of 16S rDNA by Polymerase Chain Reaction (PCR)

In this study, we used (Tamura et al., 2007) universal primer for amplification of 16S rDNA gene in all bacterial species. This primer was custom synthesized by Bangalore Genei Pvt. Ltd., Bangalore, India. The 50 μl of reaction mixture consisted of 50 ng of genomic DNA, 2.5 units of Taq polymerase, 5 μl of 10 X buffer (100 mM Tris-HCl, 500 mM KCl pH-8.3), 200 μM dNTP, 1.5 mM MgCl2 and 10 pmoles of each primer. The forward primer 27F (5′-AGAGTTTGATCCTGGCTCAG-3′) and reverse primer 1492R (5′-TACGGTTAC CTTGTT ACGACTT-3′) (Miller et al., 2013) were used. Amplification was performed under the following thermal (PCR System 2720, Applied Biosystems, Singapore) conditions: initial denaturation at 94°C for 5 min, followed by 34 cycles of denaturation at 94°C for 1 min, annealing at 52°C for 1.5 min, extension at 72°C for 2 min and a final extension at 72°C for 7 min. Amplified PCR products (5 μl) were resolved on a 1.5% (w/v) agarose gel at 100 V for 45 min in 1X TAE buffer containing ethidium bromide (EtBr) along with 500 bp DNA ladder (Bangalore Genei Pvt., Ltd. Bangalore, India). We obtained the expected size of PCR product (1500 bp). It was purified using PCR purification kit (Invitrogen, PureLink^TM^ PCR purification kit, United States) for the sequencing of 16S rDNA.

In order to establish the genetic relationship, we used 16S rDNA gene sequence of isolated species along with reference strains retrieved from ribosomal database project. Multiple sequence alignment was done by Clustal W and MEGA 4.0 software for construction of phylogenetic tree with 500 bootstrap replication. The evolutionary distances were computed using the Maximum Composite Likelihood method (Nei et al., 1985).

### Assessment of Genetic Diversity Using ISSR

Genetic diversity of the bacterial species was assessed by ISSR markers. A total 7 ISSR marker selected as it’s frequently being used in the laboratory, producing reproducible and polymorphic bands (UBC-807, UBC-808, UBC-809, UBC-811, UBC-823, UBC-824, and UBC-836) primers were used for genetic diversity assessment (**Table 6**). For ISSR PCR was performed in a volume of 15 μl reaction mixture containing 1 μl template DNA (80 ng), 1.5 μl 10 × PCR buffer, 0.35 μl of dNTPs (25 mM), 0.3 μl MgCl_2_ (1.5 mM), 1.2 μl random primer (10 pM), 0.25 μl Taq polymerase (3 units) and 10.4 μl, sterile distilled water. The reaction was carried out in a DNA Engine Dyad ALD1234 (Biorad, United States). The PCR programmed for an initial denaturation for 4 min at 94°C, then 38 cycles of 1 min denaturation at 94°C, 1 min annealing at 55°C, and 1 min extension at 72°C with a final extension at 72°C for 10 min. The PCR amplification with each ISSR primer was repeated twice to verify the reproducibility of the results. The amplified ISSR amplicons were analyzed by electrophoresis in 2.5% agarose gel using Tris–Acetic acid–Ethylene diamine tetraacetic acid buffer. The number of amplified amplicons was recorded using a gel documentation system (AlphaImager 3400).

**Table 6:** Detail of ISSR primers and their amplification profile used in genetic diversity assessment.

S. No.	ISSR	Nucleotide sequence (5′……3′)	TA (°C)	A B	S R(bp)	PB	MB	PE%	PIC	EMR	MI
1	UBC-807	AGAGAGAGAGAGAGAGT	55.0	8	300–2100	6	2	75.00	0.28	4.50	1.25
2	UBC-808	AGAGAGAGAGAGAGAGC	56.8	10	250–2250	9	1	90.00	0.40	8.10	3.24
3	UBC-809	AGAGAGAGAGAGAGAGG	58.0	7	500–1500	7	0	100.00	0.44	7.00	3.11
4	UBC-811	GAGAGAGAGAGAGAGAC	52.0	8	650–1900	7	1	87.50	0.39	6.13	2.38
5	UBC-823	TCTCTCTCTCTCTCTCC	55.0	13	300–800	12	1	92.30	0.41	11.08	4.54
6	UBC-824	TCTCTCTCTCTCTCTCG	55.0	10	250–1700	9	1	90.00	0.40	8.10	3.24
7	UBC-836	AGAGAGAGAGAGAGACYA	55.0	11	500–2000	11	0	100.00	0.44	11.00	4.89
**Performance of primer (Average)**			**–**	**9.57**	**–**	**8.71**	**0.86**	**90.69**	**0.40**	**7.99**	**3.24**

*TA, Annealing temperature; AB, Amplified bands; SR, Size range base pair; PB, Polymorphic bands; MB, Monomorphic bands; PE, Polymorphism efficiency; PIC, Polymorphism information content; EMR, Effective multiplex ratio; MI, Marker index*.

#### ISSR Data Analysis and Scoring

The amplified bands of ISSR on gels were scored as one (1) for present and zero (0) for absent. Evaluation of fragment patterns was carried out by the similarity index (SI). The similarity matrix data was calculated by Jaccard index using NTSYSpc Version 2.11X software. The polymorphism information content (PIC) were calculated by the using formula (Roldàn-Ruiz et al., 2000); PICi = 2fi (1 – fi), for each locus. Where PICi is the polymorphic information content of the locus i, fi is the frequency of the amplified fragments and 1 - fi is the frequency of non-amplified fragments. The effective multiplex ratio (EMR) is calculated by multiplying the proportion of polymorphic loci per their total number with total number of polymorphic loci (per primer) with the following formula as described by Powell et al. (1996) and Nagaraju et al. (2001) EMR = np(np/n) Where np is the number of polymorphic loci, and n is total number of loci. Marker index (MI) is a statistical parameter utilized for estimation of the total utility of the maker system. MI is the product of the PIC value (or expected heterozygosity, HE) and EMR (Powell et al., 1996; Nagaraju et al., 2001) MI = PIC ^∗^ EMR. The similarity matrix data was subjected to unweighed pair group method for arithmetic average (UPGMA) cluster analysis to generate a dendrogram using average linkage procedure.

### Effect of Different Treatment Combinations of *Bacillus* Strains on Plant Growth Attributes of *Vigna radiata* Under Plant Growth Chamber

Healthy seeds of *Vigna radiata* was selected and sterilized with 0.02% Mercuric chlorite. The sterilized seed was put on wetted cotton with sterilizing distilled water containing Petri dish for 24 h. The germination percentage of seeds was observed 98% after 24 h. We have filled 150 g sterilized garden soil in sterilized thermocol glass. The bacterial strains were grown in nutrient broth media. After seed sterilization, 10 seeds were inoculated [10^8^ colony farming unit (CFU) per seed] as per treatment combinations (**Table 7**). Ten inoculated seeds were showed in respective treatment combination of cups. The experiment was designed with 8 treatments and 3 replications. The experiment setup was put in plant growth chambers. After complete germination in each treatment, only five plants were left for further analysis. After 10 days, plants were uprooted and took length (cm/plant) and fresh weight (g/plant) of shoot and root of *Vigna radiata.*

**Table 7:** Treatment combination of bacterial strains.

S. No.	Treatments
1	Control (Un-inoculated)
2	*Bacillus subtilis* BHUJP- H1
3	*Bacillus* sp. BHUJP-H2
4	*Bacillus licheniformis* BHUJP-H3
5	*Bacillus subtilis* BHUJP- H1+ *Bacillus* sp. BHUJP-H2
6	*Bacillus subtilis* BHUJP- H1+ *Bacillus licheniformis* BHUJP-H3
7	*Bacillus* sp. BHUJP-H2+ *Bacillus licheniformis* BHUJP-H3
8	*Bacillus subtilis* BHUJP- H1+ *Bacillus* sp. BHUJP-H2+ *Bacillus licheniformis* BHUJP-H3

### Statistical Analysis

The experimental setup was prepared with nineteen treatments and three replications. The results were expressed as the mean ± SE of different independent replicates. Analysis of variance (ANOVA) followed by Duncan *post hoc* multiple comparison tests was done using SPSS software (version 16.0). The values of *P* ≤ 0.05 were considered as statistically significant.

## Result

### Morphological and Biochemical Characteristics of Thermophilic *Bacillus* Species

Three bacterial strains such as BHUJP-H1, BHUJP-H2, and BHUJP-H3 were isolated, analyzing the cultivable aerobic bacteria from hot spring and checked their morphological, biochemical properties and growth pattern on different temperature range from 30 to 60°C. The cell morphologies of *B. subtilis* species BHUJP-H1 show gram positive character, rod and circular shape, *Bacillus* sp. BHUJP-H2 reflect gram positive characteristic having circular rod and spindle form and *B. licheniformis* BHUJP-H3 show gram positive character having rod and filamentous form were observed (Verma et al., 2010). In the present study, the colonies morphology of strains BHUJP-H1 (flat elevation, curled margin, and creamy white color), BHUJP-H2 (flat elevation, undulate margin and whitish color) and BHUJP-H3 (flat elevation, curled margin and whitish color) were observed (**Table 1**). The amylase production was recorded in isolated bacterial strains BHUJP-H1 and BHUJP-H3 but not in BHUJP-H2. The biochemical characteristic such as catalase and cellulase production was observed in all three isolated *Bacillus* species. Out of which, BHUJP-H1 and BHUJP-H2 showed more cellulase production as compared to BHUJP-H3. These *Bacillus* strains were found good growth on nutrient agar media at 30, 40, 50, and 60°C. *B. subtilis* strain BHUJP-H1, *Bacillus* sp. species BHUJP-H2, and *B. licheniformis* strain BHUJP-H3 have the ability to grow in the broader temperature range from 30 to 60°C. The BHUJP-H1 was found to more growth at an extreme high temperature of 60°C as compared to BHUJP-H2 and BHUJP-H3 (**Table 2**). The *B. subtilis* BHUJP-H1 was showed faster growth rate at 60°C as compared to 30, 40, and 50°C. The variation in growth vs. temperature expressed that this bacterium can survive in adverse environmental condition.

### Plant Growth Promoting Properties of Thermophilic *Bacillus* Species

In order to estimate, indole-3-acetic acid (IAA) production was found in three thermophilic *Bacillus* strains at 150 and 300 μg/ml concentration of tryptophan, where the tryptophan is used as a precursor of IAA synthesis. The bacterial strains *Bacillus* sp. BHUJP-H2 and *B. licheniformis* BHUJP-H3 showed a significant IAA production as compared to *B. subtilis* BHUJP-H1 at concentrations (150 and 300 μg/ml) of tryptophan in broth medium after 24, 48, and 72 h incubation. IAA syntheses by bacterial strains have recorded a range from 14.65 to 34.76 μg/ml in broth medium. At the tryptophan concentration of 150 μg/ml, the IAA production was estimated significant increase in *Bacillus* sp. (25.41, 26.36, and 24.06 μg/ml) and *B. licheniformis* (20.93, 24.34, and 31.79 μg/ml) as compared to *B. subtilis* (14.65, 17.12, and 18.35 μg/ml) during incubation period of 24, 48, and 72 h, respectively. Similarly, it was also observed at 300 μg/ml concentration of tryptophan, bacterial strains *B. licheniformis* (24.55, 32.52, and 34.76 μg/ml) *Bacillus* sp. (25.74, 22.94, and 22.27 μg/ml) showed significant IAA production as compared to *B. subtilis* (21.36, 19.08, and 24.85 μg/ml) after incubation period of 24, 48, and 72 h, respectively (**Table 3**). The main entity has observed that the *Bacillus* sp. and *B. licheniformis* were found more significant IAA synthesis than *B. subtilis*.

The thermophilic *B. licheniformis* BHUJP-H3 was showed the more significant increase (33.01 μg/ml) phosphate solubilisation as compared to *Bacillus sp*. BHUJP-H2 (12.76 μg/ml) and *B. subtilis* BHUJP-H1 (12.09 μg/ml) at 3 days’ incubation in Pikovaskaya broth media at 45°C. Whereas, *Bacillus* sp. BHUJP-H2 (63.14 μg/ml) was obtained higher phosphate solubilisation followed by *B. licheniformis* BHUJP-H3 (42.14 μg/ml) as compared to *B. subtilis* BHUJP-H1 (22.48 μg/ml) at 6 days’ incubation period. *Bacillus* sp. and *B. licheniformis* BHUJP-H3 were found to be more phosphate solubilising strain than BHUJP-H1 at 3 and 6 days’ incubation, respectively (**Table 4**). Ammonia, hydrogen cyanide (HCN) and siderophore production were observed in isolated thermophilic species of *B. subtilis* and *Bacillus* sp. while *B. licheniformis* was unable to produce HCN and siderophore. *B. subtilis* was showed more HCN production as compared to others (**Table 4**).

### Insecticide Tolerance of Monocrotophos and Chlorpyrifos Ability of *Bacillus* Strains

The monocrotophos and chlorpyrifos insecticide is the commercial available group of organophosphate insecticide which is widely used in agriculture. We have tested the effect of this insecticide on microbial growth on nutrient agar media by the methods of disk diffusion. The growth inhibition test was performed by using four concentrations of insecticide 1x, 2x, 3x, and 10x. The bacterial strains BHUJP-H1, BHUJP-H2 and BHUJP-H3 were showed no inhibition zone against monocrotophos that means these strains were more tolerant (**Table 5**). While chlorpyrifos, the bacterial strains BHUJP-H1 and BHUJP-H2 showed the zone of inhibition at 1x, 2x, and 3x concentration that means these strains are susceptible. The strain BHUJP-H3 was found tolerance at all concentration of insecticides (**Table 5**), that means strains BHUJP-H3 is tolerant.

### The Molecular Characterization of Thermophilic *Bacillus*

**FIGURE 1:**
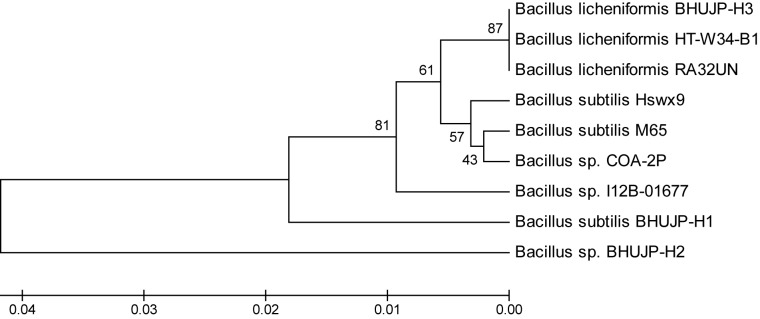
The phylogenetic tree between 9 taxa were generated using the UPGMA method. The thermophilic bacterial strains *Bacillus* sp. BHUJP-H2 and *B. subtilis* BHUJP-H1 is more closely related taxa as compared *B. licheniformis* BHUJP-H3. BHUJP-H3 showed similarity with known taxa of HT-W34-B1 and RA32UN.

The Genomic DNA of all species was resolved on 0.8% agarose gel. The 16S rDNA was amplified by PCR using 16S universal primers 27F forward and 1492R reverse primer. Amplified 16S rDNA gene was resolved on the agaroge gel and their size was found to be 1500 bp. It was sequenced by automated sequencer from Centre of Human Genetic Disorder, Institute of Science, BHU, Varanasi, India. The 16S rDNA analysis followed by BLAST search exhibited close to 16S rDNA database similarity. A comparison with the 16S rDNA sequences available in the GenBank database indicated that the strain BHUJP-H1, showed 98% similarity with *B. subtilis* strain M65 (Accession No. KC315772). The strain BHUJP-H2 showed 93% similarity with *Bacillus* sp. COA-2P (Accession no. KM575935) and also strain BHUJP-H2 showed 99% similarity with *B. licheniformis* strain RA32UN (Accession No. KJ867517). According to similarity index of the partial gene sequence, the microbial strains BHUJP-H1, BHUJP-H2 and BHUJP-H3 were confirmed microbial strains as *Bacillus* genus. The sequences of the strains BHUJP-H1, BHUJP-H2 and BHUJP-H3 were deposited in NCBI GenBank database with different accession number KU312403, KU312404, and KU312405, respectively. The 16S rDNA has confirmed the identification of hot spring bacteria such as *B. subtilis* strain BHUJP-H1 (Accession No. KU312403), *Bacillus* sp. strain BHUJP-H2 (Accession No. KU312404) and *B. licheniformis* strain BHUJP-H3 (Accession No. KU312405). The phylogenetic tree between 9 taxa were generated using the UPGMA method (**Figure 1**). The thermophilic bacterial strains *Bacillus* sp. BHUJP-H2 and *B. subtilis* BHUJP-H1 is more closely related taxa as compared *B. licheniformis* BHUJP-H3. BHUJP-H3 showed similarity with known taxa of HT-W34-B1 and RA32UN.

### Genetic Diversity Assessment of Thermophilic *Bacillus* by ISSR

**FIGURE 2:**
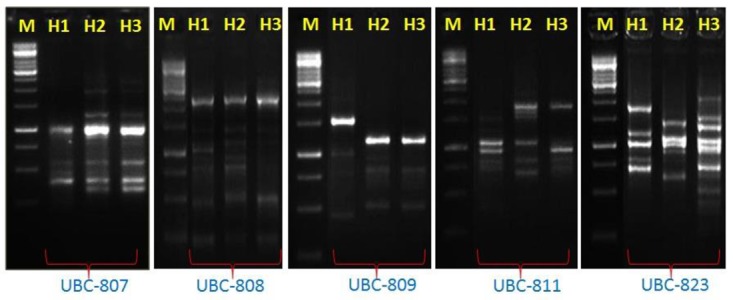
Inter simple sequence repeat (ISSR) analysis of three *Bacillus* strains by using 7 sets of primers: UBC-807, UBC-808, UBC-809, UBC-811, UBC-823, UBC-824, and UBC-836, here gel picture of primers UBC-807, UBC-808, UBC-809, UBC-811, and UBC-823 showed different band pattern on 2.5% gel agarose gel M: Marker size 1 kb plus (Banglore genei), amplification between three strain H1: *B. subtilis* BHUJP-H1; H2: *Bacillus* sp. BHUJP-H2 and H3: *B. licheniformis* BHUJP-H3.

In order to assess the genetic diversity, the ISSR have been performed among these species. The genomic DNA of thermophilic *B. subtilis* BHUJP-H1, *Bacillus* sp. BHUJP-H2 and *B. licheniformis* BHUJP-H3 has PCR amplified gene in respect of seven primers. Out of all screened primers, UBC-809, UBC-836, shows 100% polymorphism efficiency while UBC-823 shows 92.30%, UBC-808 and UBC-824 shows 90.00%, UBC-811 and UBC-807 showed 87.50 and 75.00% polymorphism respectively and overall polymorphism efficiency was 90.69% (**Table 6**). The average number of polymorphic band amplified by ISSR was 8.71 per primer and the number of amplicons varies from 7 to 13 with a size range of 250-2250 base pair. The highest polymorphism content (PIC) showed by the UBC-809 and UBC-836 while the lowest, 0.28 by UBC-807, the overall average PIC 0.40 obtained (**Table 6**). The highest, 11.08 and lowest, 4.50 EMR obtained for primer UBC-823 and UBC-807, the average EMR of the used primers, 7.99 obtained (**Table 6**). The highest, 4.89 and lowest, 1.25 MI obtained with primer UBC-836 and UBC-807 respectively and an average of 3.24 MI obtained with all used primer. The amplified bands showed similar banding pattern with respect to seven random primers between thermophilic *B. subtilis* BHUJP-H1 and *Bacillus* sp. BHUJP-H2 Whereas, *B. licheniformis* BHUJP-H3 showed different banding patterns that indicate this strain is genetically different from BHUJP-H1 and BHUJP-H2 (**Figure 2**). In dendrogram, *B. subtilis* BHUJP-H1 and *Bacillus* sp. BHUJP-H2 grouped under the same cluster whereas *B. licheniformis* BHUJP-H3 in separate cluster. The ISSR analysis showed high polymorphism among the three isolates.

**FIGURE 3:**
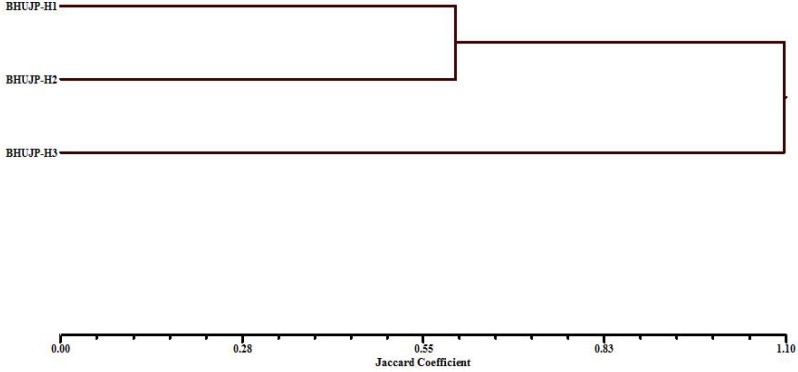
UPGMA dendrogram based on the Nei’s genetic similarity index illustrating the genetic relationship among 3 *Bacillus* species.

The genetic diversity between isolated thermophilic *B. subtilis* BHUJP-H1, *Bacillus* sp. BHUJP-H2 and *B. licheniformis* BHUJP-H3 was done by ISSR profile, which classified them into two groups based on their capacity for producing various biochemical and plant growth promoting activities (**Figure 3**).

### Effect of Different Treatment Combinations of *Bacillus* Strains on Plant Growth Attributes of *Vigna radiata*

**FIGURE 4:**
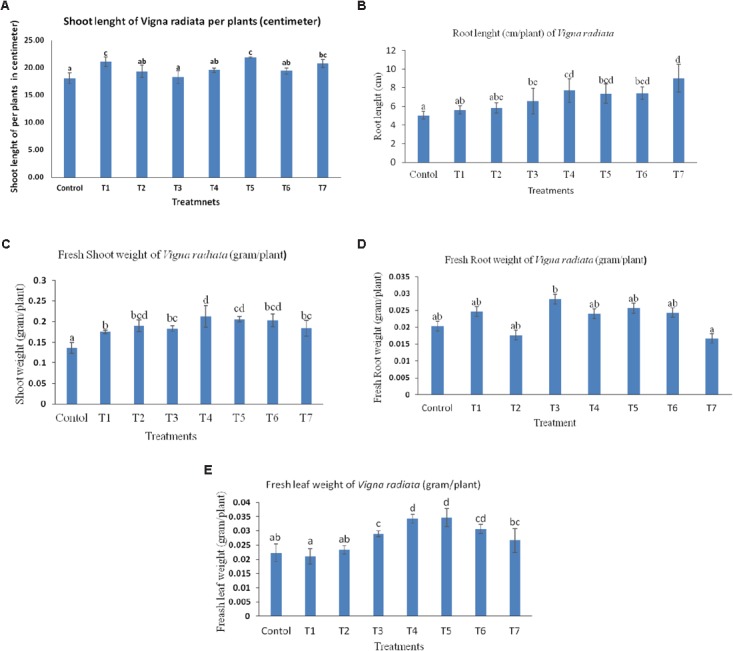
Effect of microbial culture on plant growth attributes after 10 days inoculation, **(A)** shoot length (cm) of *Vigna radiate* per plant; **(B)** Root length (cm) of *Vigna radiata* per plant; **(C)** fresh shoot weight (g) of *Vigna radiata* per plant; **(D)** Fresh root weight (g) of *Vigna radiata* per plant; **(E)** Fresh leaf weight (g) of *Vigna radiata* per plant.

The treatment combination of *B. subtilis* BHUJP- H1, *B. subtilis* BHUJP- H1+ *B. licheniformis* BHUJP-H3, and *B. subtilis* BHUJP- H1+ *Bacillus* sp. BHUJP-H2+ *B. licheniformis* BHUJP-H3 were recorded significant growth of shoot length (cm/plant) as compared to control (Un-inoculated) and others after 10 days seedling growth of mungbean plants (**Table 7**). The *B. subtilis* BHUJP- H1+ *B. licheniformis* BHUJP-H3 and *B. subtilis* BHUJP- H1 were found the more significant increase in shoot length as compared to others (**Figure 4A**). In root length, treatment *B. subtilis* BHUJP-H1+ *Bacillus* sp. BHUJP-H2+ *B. licheniformis* BHUJP-H3 was found more significant enhancement followed by *B. subtilis* BHUJP-H1+*Bacillus* sp. BHUJP-H2, *B. subtilis* BHUJP-H1+ *B. licheniformis* BHUJP-H3 and *Bacillus* sp. BHUJP-H2+ *B. licheniformis* BHUJP-H3 than control, *B. subtilis* BHUJP- H1, *Bacillus* sp. BHUJP-H2 and *B. licheniformis* BHUJP-H3 (**Figure 4B**).

**FIGURE 5:**
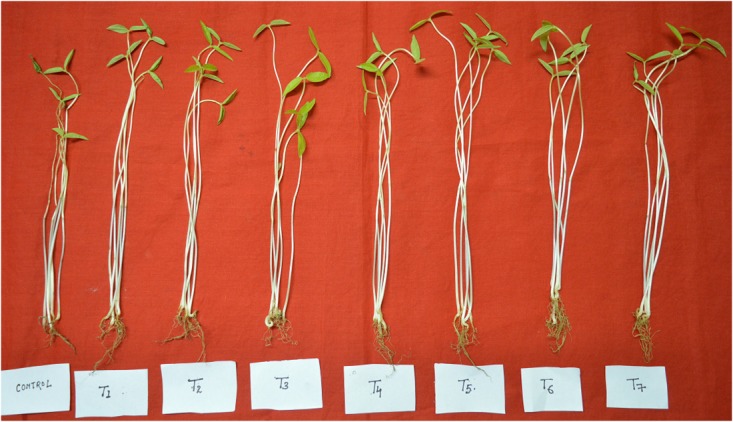
Effect of microbial inoculation of enhancing plant height as per different treatment combination of *Vigna radiata* after 10 days seed inoculation.

The fresh shoot weight of mungbean was recorded after 10 days uprooted plants of different treatment combination. Fresh shoot weight was significantly observed more in all treatment combinations as per controls. Treatment *B. subtilis* BHUJP-H1+ *Bacillus sp.* BHUJP-H2 and *B. subtilis* BHUJP-H1+ *B. licheniformis* BHUJP-H3 were found the more significant fresh weight of shoot followed by *Bacillus* sp. + *B. licheniformis, Bacillus* sp. BHUJP-H2, *B. subtilis* + *Bacillus* sp. + *B. licheniformis*, *B. licheniformis* and *Bacillus* sp. as compared to control (**Figure 4C**). But in the case of root weight (g/plant) was found only significant in treatment *B. licheniformis* BHUJP-H3 as compared to all others while the enhancement of root weight was observed in treatment combination of strains BHUJP-H1+ BHUJP-H2, BHUJP- H1+ BHUJP-H3, BHUJP-H2+BHUJP-H3 and BHUJP-H1 as compared to others (**Figure 4D**). The leaf fresh weight (g/plant) was recorded more significant in treatment combination of *B. subtilis* BHUJP- H1+ *B. licheniformis* BHUJP-H3 and *B. subtilis* BHUJP- H1+ *Bacillus* sp. BHUJP-H2 followed by *Bacillus* sp. BHUJP-H2+ *B. licheniformis* BHUJP-H3, *B. licheniformis* BHUJP-H3 and *B. subtilis* BHUJP- H1+ *Bacillus* sp. BHUJP-H2+ *B. licheniformis* BHUJP-H3 as compared to control after 10 days seedling growth (**Figure 4E**). Overall, an impact of different treatment combination of *Bacillus* strains has enhanced the shoot length and fresh shoot weight as compared to control (**Figure 5**).

## Discussion

### Bacterial Screening and Biochemical Properties

There is increasing the global warming and climate changes, a major issue that affects the biodiversity. In the present study, we isolated bacteria from extreme hot spring condition so they have very good properties for producing thermo-tolerant enzymes and novel biomolecules (plant growth promoter compound) that can play a key role for industrial, agricultural and biotechnological applications. The selected species were identified as *Bacillus* by comparing with the reference strains of *B. subtilis* BHUPSB13, *Paenibacillus polymyxa* BHUPSB16 (Verma et al., 2016) and *B. megaterium* BHUPSB14 (Verma and Yadav, 2018). As per morphological, biochemical and molecular characterization, the isolated bacterial strains were identified as *B. subtilis* BHUJP-H1, *Bacillus* sp. BHUJP-H2 and *B. licheniformis* BHUJP-H3. The current study revealed that *Bacillus* strains were found in the sediment of hot spring of Chumathang area, Ladakh region. Previously, Yadav et al. (2015) have studied about a microbial diversity of various hot spring water and sediments that they found *B. megaterium, B. subtilis, B. firmus*, and *B. pumilus*.

The amylase, catalase, and cellulase production were observed in BHUJP-H1, BHUJP-H2 and BHUJP-H3, while straining BHUJP-H2 unable to produce amylase. These enzymes are thermostable and capable of producing various types of enzymes for industrial use and these strains can be used as the future alternative of enzymes production. Also, identifying enzymes producing capability these extremophilic stains are required to access for utility in various fields of medicine, agriculture and microbiology. The characteristic, especially, cellulase enzyme plays a key role in the degradation of cellulosic material for bioethanol production and also degradation of organic residues for enhancing the soil fertility and health. The cellulase enzyme play an important role in composting of agro-cellulosic material in soil to enhance soil fertility and health by providing adequate carbon source for survival of rhizosphere microbes and their proliferation. The catalase is also used for the manufacture of baked goods, beverages preparation, the textile industry and cosmetic industry (Zhang et al., 2010). Amylase also plays a significant role in starch degradation (Abd-Elhalem et al., 2015). *B. subtilis* BHUJP-H1, *Bacillus* sp. strain BHUJP-H2, and *B. licheniformis* strain BHUJP-H3 has the ability to grow in a wide range of temperatures (30–60°C). The variation in growth temperature (30, 40, 50, and 60°C) expressed that this can survive in adverse environmental condition. A number of studies show that is possible because of the bacterial genome have been modified in a stress condition for better survival and fitness in changing environmental conditions. It may be possible to bacterial genes could be regulated and adjusted according to adverse conditions (Panda et al., 2015). Thermophilic microorganisms have recently gained more scientific and industrial importance because of their thermal stability and activity (Coolbear et al., 1992). Similarly, Yadav et al. (2015) have reported that the extreme conditions may be resources for the new genus of *Bacillus* that can be flourished well under the extremes pH, temperature, salinity, and moisture. *Bacillus* strains have the ability to produce endospore that can protect them from the diverse environmental condition.

### Plant Growth Promoting Properties of *Bacillus* Strains

Plant growth promoting properties of *B. subtilis* BHUJP-H1, *Bacillus* sp. BHUJP-H2 and *B. licheniformis* BHUJP-H3 were showed production of IAA, HCN, ammonia and siderophore and the phosphate solubilisation except for the BHUJP-H3 that unable to produce HCN and siderophore. In the previous study, *B. subtilis*, IAA production was found more significantly increased in *Bacillus* sp. and *B. licheniformis* according to increase the concentration of tryptophan. Similarly, Ahmad et al. (2005) have also observed that *Azotobacter* and *Pseudomonas* show the ability for enhancing the IAA production according to increase in the concentration of tryptophan, where tryptophan uses a precursor for IAA synthesis under the broth culture. IAA concentration may vary between the different strains and it is also affected by growth, media and nutrient availability (Sridevi and Veera Mallaiah, 2007). IAA plays a vital role in the plant growth hormones for enhancing the cell division, plant growth, and yield (Verma et al., 2010). *Bacillus* sp. was observed more significant IAA production followed by *B. licheniformis* than the *B. subtilis*. Swain et al. (2007) have previously reported IAA producing by *B. subtilis* spp. That showed significantly higher than *Burkholderia* sp., *Bacillus* sp., *Pseudomonas* sp. BHUPSB04, *Pseudomonas* sp. BHUPSB06 and *Paenibacillus* sp. as compared to *Trichoderma* and *Azospirillum* under the broth culture in presence of tryptophan (Verma et al., 2010, 2014). *Bacillus* sp. BHUJP-H2 and *B. licheniformis* BHUJP-H3 has recorded more significant enhancement in the phosphate solubilisation than the Bacillus sp. BHUJP-H1. Phosphate solubilisation occurs by *Bacillus* species because of production of the acids for lowering the pH of broth media that support the conversion of an insoluble form of phosphate into the soluble form. These strains can be used as effective and efficient phosphate solubilising strains for enchanting the agricultural productivity. In previous reports by Rudresh et al. (2005), Valverde et al. (2006), Ahmad et al. (2008), and Verma et al. (2010, 2014) have shown that *Pseudomonas, Bacillus*, *Azotobacter*, and *Mesorhizobium* are potential phosphate solubilizers. In the present study, ammonia production was shown by all isolated strains whereas, the HCN and siderophore estimated by BHUJP-H1 and BHUJP-H2. Ammonia is significantly effective for plant growth and also the enhancing soil fertility. Whereas, the HCN and siderophore were estimated by BHUJP-H1 and BHUJP-H2. Siderophore provides the iron for plant growth. HCN production indirectly helps in plant growth by suppressing the growth of soil borne-phytopathogens and it blocked the electron chain in pathogens for decreasing the population. *Bacillus* sp. BHUJP-H1, *Bacillus* sp. BHUJP-H2 and *B. licheniformis* BHUJP-H3 show the strong ability of plant growth promoting properties. It can provide nutrients to crops by the direct mechanism including these plant growth properties like IAA, ammonia for nitrogen, siderophore for the iron and solubilisation of phosphate for phosphorus whereas, the indirect mechanism such as HCN production to suppress the plant pathogens.

### Effect of Monocrotophos and Chlorpyrifos Insecticide on Growth of *Bacillus* Strains

The monocrotophos and chlorpyrifos insecticide are a commercially available group of organophosphate insecticide which are the broad range of insecticide used in agricultural production. The bacterial strains BHUJP-H1, BHUJP-H2 and BHUJP-H3 were showed no inhibition zone against monocrotophos that means these strains are more tolerant. The strains BHUJP-H1 and BHUJP-H2 showed a zone of inhibition at 1x, 2x, and 3x concentration chlorpyrifos and these strains are more susceptible. While strain BHUJP-H3 was found tolerance at all concentration of insecticides. The tolerant strain can have the ability to degrade or resistance against monocrotophos and chlorpyrifos while the others strains but some strains have shown growth inhibition zone at a different concentration, these types stains may not have the ability to degrade the insecticides. Those strains may be degraded the insecticide and used as a sole source of carbon for their growth and development (Verma et al., 2014; Hamada et al., 2015; Jadhav and David, 2016).

### Molecular Identification by 16S rDNA and Diversity Assessment of Thermophilic *Bacillus* by ISSR

The partial 16S rDNA gene sequencing, thermophilic bacterial strains BHUJP-H1, BHUJP-H2 and BHUJP-H3 were identified as Bacillus sp. BHUJP-H1 (KU312403), *B. subtilis* BHUJP-H2 (KU312404) and *B. licheniformis* BHUJP-H3 (KU312405). Yadav et al., 2015 have previously reported that 13 genera, 9 (*Bacillus, Halobacillus, Lysinibacillus, Oceanobacillus, Paenibacillus, Salinibacillus, Sediminibacillus, Thalassobacillus*, and *Virgibacillus*) belong to Bacillaceae and 4 (*Ammoniphilus, Aneurinibacillus, Brevibacillus*, and *Paenibacillus*) that belongs to Paenibacillaceae were isolated from thermal plants. Similarly, Acharya and Chaudhary (2012) isolated from Barkeshwar hot spring in West Bengal and identified as *B. licheniformis* WBS1 and *Bacillus* sp. WBS3 by 16S rRNA. The 16S rDNA is easy tools for identification of microorganisms using BLAST. Similar sequences of known bacterial strains were identified for construction of the phylogenetic tree. The phylogenetic tree between 9 taxa was constructed by using UPGMA. The thermophilic BHUJP-H3 showed similarity with the known taxa of strains HT-W34-B1 and RA32UN.

Inter simple sequence repeat markers are one of the cheapest and easiest marker systems with high efficiency in generating polymorphism among populations and being a PCR based fingerprint, very helpful and informative tool in genetic diversity studies as well as it is also a fast genotyping technique which is widely used in characterization of genetic diversity among populations (Baysal et al., 2011; Akramipour et al., 2017). In the present study the genetic diversity assessment performed by ISSR molecular markers which is generally used in microbial diversity assessment (Baysal et al., 2011; Baysal, 2015; Rayar et al., 2015; Akramipour et al., 2017). Total 7 ISSR primers used for assessment produced 7-13 bands and 67 total bands out of which 61 polymorphic bands with an average 8.71 bands per primer and 90.68 overall polymorphism efficiency. The PIC, the ability of a marker to establish polymorphism in the population depending on the number of alleles detected and on their distribution frequency (Botstein et al., 1980). Thus, PIC identifies the discriminatory ability of the marker. In present study the highest and the lowest PIC obtained by the UBC-809 and UBC-836 while the lowest, 0.28 the overall PIC value 0.40 obtained by the used marker system (**Table 6**) suggest high polymorphism among the species. For the dominant markers, the maximum PIC value is 0.5. The markers having similar distribution in population higher the PIC values. The PIC value also depends on the distribution frequency of the alleles (Chesnokov and Artemyeva, 2015). The total number of polymorphic loci (per primer) is the measure of EMR, the higher EMR value, higher the effectiveness of the primer marker system. In the present study the highest, 11.08 and lowest, 4.50 EMR obtained for primer UBC-823 and UBC-807, the average EMR value for the used primers, 7.99 obtained (**Table 6**). The high EMR suggest that the used ISSR markers are potential for the study of genetic diversity within bacterial population, our result is also supported by recent study where the ISSR markers potentially used for assessment of genetic diversity (Baysal et al., 2011; Rayar et al., 2015; Akramipour et al., 2017). The MI is a statistical factor which estimate whole effectiveness of the used maker system. The higher MI value indicate better is the method (Nagaraju et al., 2001; Chesnokov and Artemyeva, 2015). In present study the highest, 4.89 and lowest, 1.25 MI obtained with primer UBC-836 and UBC-807 respectively and with an average of 3.24 MI obtained with all used primer. The high MI value proved the suitability of ISSR marker for genetic assessment in bacterial population, the result is supported by the recent (Baysal et al., 2011; Rayar et al., 2015; Akramipour et al., 2017) findings. The ISSR assessment showed that the thermophilic *B. subtilis* BHUJP-H1 and *Bacillus* sp. BHUJP-H2 showed similar band pattern. While, *B. licheniformis* BHUJP-H3 showed different banding patterns which means this strain is genetically different from strains BHUJP-H1 and BHUJP-H2. The dendrogram was constructed by UPGMA method based to find out that the *B. subtilis* BHUJP-H1 and *Bacillus* sp. BHUJP-H2 showed the close relationship between them as compared to *B. licheniformis* BHUJP-H3. Our result also showed that all seven ISSR markers worked efficiently in genetic diversity assessment. The genetic diversity of *B. subtilis* BHUJP-H1, *Bacillus* sp. BHUJP-H2 and *B. licheniformis* BHUJP-H3 were done by ISSR profile, which classified them in two groups according to their capacity for producing various biochemical and plant growth promoting properties.

In the present study, the cluster I showed only single *B. licheniformis* BHUJP-H3 and cluster II showed two species *B. subtilis* BHUJP-H1 and *Bacillus* sp. BHUJP-H2. In both clusters, some biochemical and plant growth promoting activities were found to significant difference including IAA production, phosphate solubilisation, and ammonia while HCN and siderophore production only produced in the cluster I which belongs to group of *Bacillus* strains BHUJP-H1 and BHUJP-H2. The genetic polymorphism of *Bacillus* strains from hot spring sources by RAPD and phenotypic characteristic has been studied by Hazem and Manar (2003) and resulted in 5 major clusters with the 60% similarity. The thermophilic strains have the ability to play a key role for industrial applications. Furthermore, *Bacillus* sp. BHUJP-H1, *Bacillus* sp. BHUJP-H2 and *B. licheniformis* BHUJP-H3 can be used as drought resistance plant growth promoting strains for sustainable agricultural production. Also, these strains can be used for harnessing some industrially important enzymes because of improving the growth under the extreme environmental conditions. It can be useful for maintaining the soil health and early warning indicators of environmental changes, it became essential to study its genetic diversity.

### Effect of Bio-inoculant of *Bacillus* Strains on Plant Growth Attributes of *Vigna radiata* Under Plant Growth Chamber

Plant growth of any plants was affected by soil nutrient content which is totally governed by the different types of microbes and their physiological and biological process to help in enhancing soil fertility and health under rhizosphere. The healthy rhizosphere plays an important role in enhancing the plant growth by the direct indirect mechanism of soil microbes Verma et al. (2010). We have attempted to take different treatment combination of plant growth promoting bacillus strains for enhancing plant growth attributed under plant growth chamber. The significant enhancement of shoot length (cm/plant) in treatment combination of *B. subtilis* BHUJP-H1, *Bacillus* sp. BHUJP-H2, *B. subtilis* BHUJP-H1+ *Bacillus* sp. BHUJP-H2, *B. subtilis* BHUJP-H1+ *B. licheniformis* BHUJP-H3, *Bacillus* sp. BHUJP-H2+ *B. licheniformis* BHUJP-H3 and *B. subtilis* BHUJP-H1+ *Bacillus* sp. BHUJP-H2+ *B. licheniformis* BHUJP-H3 were recorded as compared to control (Un-inoculated) and *Bacillus licheniformis* BHUJP-H3 after 10 days seed inoculation. The combination *B. subtilis* BHUJP-H1+ *B. licheniformis* BHUJP-H3, *B. subtilis* BHUJP-H1 and *B. subtilis* BHUJP-H1+ *Bacillus sp.* BHUJP-H2+ *B. licheniformis* BHUJP-H3 were found a more significant increase in shoot length followed by others, because the *B. subtilis* BHUJP-H1 and *B. licheniformis* BHUJP-H3 produce more IAA which help as plant growth hormones to promote shoot growth as compared to *Bacillus* sp. BHUJP-H3. While the strain *B. subtilis* BHUJP-H1 was not enhanced significant growth of root length but root length recorded higher than control. The possibility of non-significant root length growth by *Bacillus subtilis* BHUJP-H1 can be very short experiment in small cup or may be production of HCN can be inhibit the growth of root length. Some studies by Bakker and Schippers (1987) and Bakker et al. (1989) have been reported that the *Pseudomonas* spp. suspected to inhibit potato root development by their production of hydrogen cyanide. Cyanide producing *Pseudomonas* spp. also causes growth inhibition in lettuce and bean (Alstrom and Burns, 1989; Schippers et al., 1990).

Others, strains BHUJP-H3 and BHUJP-H3 showed maximum phosphate solubilisation and ammonia production while *Bacillus subtilis* BHUJP-H1 showed average phosphate solubilisation, Ammonia, HCN, and siderophore. These parameters may be support for enhancing shoot length of mungbean plant in soils under plant growth chambers. The combination of BHUJP-H1+ BHUJP-H2+ BHUJP-H3, BHUJP-H1+ BHUJP-H2, BHUJP-H1+ BHUJP-H3, and BHUJP-H2+ BHUJP-H3 were recorded more significant increase in root length as compared to control. Fresh shoot weight was significantly observed more in all treatment combinations as per control. The treatment *Bacillus licheniformis* BHUJP-H3 gave only significant enhancement of root weight (g/plant) than others while the enhancement of root weight was observed in others treatment combination. The leaf fresh weight (g/plant) was recorded more significant in treatment combination of BHUJP-H1+ BHUJP-H3 and BHUJP-H1+ BHUJP-H2, BHUJP-H1+ BHUJP-H2+ BHUJP-H3 as compared to control after 10 days seedling growth. Overall, the impact of different treatment combination of *Bacillus* strains has enhanced the shoot length and fresh shoot weight as compared to control. Similarly, the enhancement of plant growth was found due to plant growth promoting properties of different strains. Ait Kaki et al. (2017) reported that *B. amyloliquefaciens* (4RH) strain showed very significant property of biocontrol and biofertilization characteristics under *in vitro* so he recommended a potential agent for future bioinsecticide for integrate pest management and organic agricultural productions. Figueiredo et al. (2008) have been found that CIAT 899 rhizobia strains co-inoculated with *Paenibacillus polymyxa* strain DSM 36 which enhance higher shoot and root dry weight than single inoculation with CIAT 899 strain in common bean. Similarly, Elkoca et al. (2010) reported an increased shoot dry weight as a result of co-inoculation of common bean with *B. megaterium* (M-3) strain and *Rhizobium* strain. The treatment combination of *B. subtilis* BHUJP-H1, *B. subtilis* BHUJP-H1+ *B. licheniformis* BHUJP-H3 and *B. subtilis* BHUJP-H1+ *Bacillus* sp. BHUJP-H2+ *Bacillus licheniformis* BHUJP-H3 were recorded more better combination for enhancing plant growth attributes of *Vigna radiata* followed by *B. subtilis* BHUJP-H1+ *Bacillus* sp. BHUJP-H2 and *Bacillus* sp. BHUJP-H2+ *B. licheniformis* BHUJP-H3 as compared to control and others.

## Conclusion

The optimal growth of strain BHUJP-H1 was at the extreme temperature of 60°C as compared to BHUJP-H2 and BHUJP-H3. BHUJP-H1 was found efficient growth at temperature 60°C than 30, 40, and 50°C. *Bacillus* sp. BHUJP-H1, *Bacillus* sp. BHUJP-H2 and *B. licheniformis* BHUJP-H3 were produced catalase, cellulase, ammonia, HCN, siderophore, IAA and also solubilised the phosphate, while *B. licheniformis* BHUJP-H3 could not be produced HCN and siderophore. Strain BHUJP-H1, *Bacillus* sp. BHUJP-H2 and *B. licheniformis* BHUJP-H3 gave good cellulase activities and tolerant against monocrotophos insecticide while straining BHUJP-H1 and BHUJP-H2 showed susceptible against chlorpyrifos at 1x, 2x, and 3x concentration. The treatment combination of *B. subtilis* BHUJP-H1, *B. subtilis* BHUJP-H1+ *B. licheniformis* BHUJP-H3 and *B. subtilis* BHUJP-H1+ *Bacillus* sp. BHUJP-H2+ *B. licheniformis* BHUJP-H3 were recorded better combination for enhancing plant growth attributes of *Vigna radiata* followed by as compared to control and others. The stains *B. subtilis* BHUJP-H1, *B. subtilis* BHUJP-H1+ *B. licheniformis* BHUJP-H3, and *B. subtilis* BHUJP-H1+ *Bacillus* sp. BHUJP-H2+ *B. licheniformis* BHUJP-H3 can be further used as effective microbial inoculant for enhancing production of mungbean under field conditions. Others strain *Bacillus* sp. BHUJP-H1 and *Bacillus* sp. BHUJP-H2 can be used as drought tolerant plant growth promoting bacteria for enhancing the sustainable agriculture production. In future, these strains can be used as a consortium for drought tolerant bio-inoculants for agricultural farming.

## Author Contributions

JV wrote and edited the manuscript as well as made design experiment. DJ did all the experiment from microbial isolation to their properties and plant growth analysis. SP provided the soil samples for microbial isolation, helped with editing, and provided suggestions for the experiment. JY provided the lab facility for microbial isolation, helped with editing and the experiment designing. VS helped with manuscript editing and provided suggestions for molecular diversity. RK helped with molecular diversity analysis of bacterial strains by ISSR primers and wrote this art in manuscript.

## Conflict of Interest Statement

The authors declare that the research was conducted in the absence of any commercial or financial relationships that could be construed as a potential conflict of interest.

## References

[B1] Abd-Elhalem B. T. El-Sawy M. Gamal R. F. Abou-Taleb K. A. (2015). Production of amylases from *Bacillus amyloliquefaciens* under submerged fermentation using some agro-industrial by-products. *Ann. Agric. Sci.* 60 193–202. 10.1016/j.aoas.2015.06.001

[B2] Acharya S. Chaudhary A. (2012). Alkaline cellulase produced by a newly isolated thermophilic *Aneurinibacillus thermoaerophilus* WBS2 from hot spring, India. *Afr. J. Microbiol. Res.* 6 5453–5458.

[B3] Ahmad F. Ahmad I. Khan M. S. (2005). Indole acetic acid production by the indigenous isolates of *Azotobacter* and fluorescent *Pseudomonas* in the presence and absence of tryptophan. *Turkish J. Biol.* 29 29–34.

[B4] Ahmad F. Ahmad I. Khan M. S. (2008). Screening of free-living rhizospheric bacteria for their multiple plant growth promoting activities. *Microbiol. Res.* 163 173–181. 10.1016/j.micres.2006.04.001 16735107

[B5] Ait Kaki A. Kara Ali M. Milet A. Moula N. Thonart P. Kacem Chaouche N. (2017). In vitro biocontrol and biofertilization features study of a *Bacillus amyloliquefaciens* (4RH) strain isolated from a hot spring soil in Algeria. *Afr. J. Microbiol. Res.* 11 1564–1572. 10.5897/AJMR2017.8745

[B6] Akramipour N. Abdollahi M. Taghavi S. M. Rezaei R. (2017). Genetic diversity of soft rot strains of *Pectobacterium*, isolated from different hosts, using ISSR marker. *Arch. Phytopathol. Plant Prot.* 50 789–801. 10.1080/03235408.2017.1384195

[B7] Alstrom S. Burns R. G. (1989). Cyanide production by rhizobacteria as a possible mechanism of plant growth inhibition. *Biol. Fertil. Soils* 7 232–238. 10.1007/BF00709654

[B8] Aneja K. R. (2003). *Experiments in Microbiology, Plant Pathology and Biotechnology*, 4th Edn. Daryaganj: New Age International Publishers.

[B9] Bakker A. W. Bakker P. A. H. M. Schippers B. (1989). “Deleterious cyanide-producing rhizosphere pseudomonads as a factor limiting potato root growth and tuber yield in high frequency potato-cropping soil,” in *Effects of Crop Rotation on Potato Production in the Temperate Zones*, eds Vos J. Loon van C. D. Bollen G. J. (Dordrecht: Kluwer Academic Publishers), 153–162.

[B10] Bakker A. W. Schippers B. (1987). Microbial cyanide production in the rhizosphere in relation to potato yield reduction and *Pseudomonas* spp.-mediated plant growth stimulation. *Soil Biol. Biochem.* 19 249–256. 10.1016/0038-0717(87)90037-X

[B11] Bauer A. W. Kirby W. M. M. Sherris J. C. Turck M. (1966). Antibiotic susceptibility testing by a standardized single disk method. *Am. J. Clin. Pathol.* 45 493–496. 10.1093/ajcp/45.4_ts.493 5325707

[B12] Baysal Ö. (2015). “Host resistance: SAR and ISR to plant pathogenic bacteria,” in *Sustainable Approaches to Controlling Plant Pathogenic Bacteria*, eds Kannan V. Bastas K. (Boca Raton, FL: CRC Press), 205–222. 10.1201/b18892-11

[B13] Baysal Ö. Mercati F., İkten H. Yıldız R. Ç Carimi F. Aysan Y., (2011). *Clavibacter michiganensis* subsp. *michiganesis*: tracking strains using their genetic differentiations by ISSR markers in Southern Turkey. *Physiol. Mol. Plant Pathol.* 75 113–119. 10.1016/j.pmpp.2010.10.002

[B14] Belkova N. L. Tazaki K. Zakharova J. R. Parfenova V. V. (2007). The activity of bacteria in water of hot springs from Southern and Central Kamchatskaya geothermal provinces, Kamchatka Peninsula, Russia. *Microbiol. Res.* 162 99–107. 10.1016/j.micres.2006.01.006 16546359

[B15] Botstein D. White R. L. Skolnick M. Davis R. W. (1980). Construction of a genetic linkage map in man using restriction fragment length polymorphisms. *Am. J. Hum. Genet.* 32 314–331. 6247908 PMC1686077

[B16] Bric J. M. Bostock R. M. Silverstone S. E. (1991). Rapid in situ assay for indole acetic acid production by bacteria immobilized on nitrocellulose membrane. *Appl. Environ. Microbiol.* 57 535–538. 16348419 10.1128/aem.57.2.535-538.1991 PMC182744

[B17] Cappuccino J. G. Sherman N. (1992). *Microbiology, a Laboratory Manual*, 3rd Edn. New York, NY: Benjamin/Cummings Publication Co, 125–179.

[B18] Chesnokov Y. V. Artemyeva A. M. (2015). Evaluation of the measure of polymorphism information of genetic diversity. *Agric. Biol.* 50 571–578.

[B19] Cihan A. C. Tekin N. Ozcan B. Cokmus C. (2012). The genetic diversity of genus *Bacillus* and the related genera revealed by 16S rRNA gene sequences and ardra analyses isolated from geothermal regions of turkey. *Braz. J. Microbiol.* 43 309–324. 10.1590/S1517-838220120001000037 24031834 PMC3768990

[B20] Claus D. Fritz D. (1989). “Taxonomy of bacillus,” in *Bacillus*, ed. Harwood C. R. (New York, NY: Plenum Press), 5–26.

[B21] Coolbear T. Daniel R. Morgan H. W. (1992). The enzymes from extreme thermophiles, bacterial sources, thermos ability and industrial relevance. *Adv. Biochem. Eng. Biotechnol.* 45 57–98. 10.1007/BFb0008756 1605092

[B22] Elkoca E. Turan M. Donmez M. F. (2010). Effects of single, dual and triple inoculations with *Bacillus subtilis*, *Bacillus megaterium* and *Rhizobium leguminosarum* BV. *phaseoli* on nodulation, nutrient uptake, yield and yield parameters of common bean (*Phaseolus vulgaris* L. CV. ‘ELKOCA-05’). *J. Plant Nutr.* 33 2104–2119. 10.1080/01904167.2010.519084

[B23] Figueiredo M. V. B. Martinez C. R. Burity H. A. Chanway C. P. (2008). Plant growth-promoting rhizobacteria for improving nodulation and nitrogen fixation in the common bean (*Phaseolus vulgaris* L.). *World J. Microbiol. Biotechnol.* 24 1187–1193. 10.1007/s11274-007-9591-4

[B24] Ghati A. Sarkar K. Paul G. (2013). Isolation, characterization and molecular identification of esterolytic thermophilic bacteria from an Indian hot spring. *Curr. Res. Microbiol. Biotechnol.* 1 196–202.

[B25] Ghosh D. Bal B. Kashyap V. K. Pal S. (2003). Molecular phylogenetic exploration of bacterial diversity in a Bakreshwar (India) hot spring and culture of Shewanella related thermophiles. *Appl. Environ. Microbial.* 69 4332–4336. 10.1128/AEM.69.7.4332-4336.2003 12839826 PMC165147

[B26] Gilbert J. J. Jack J. D. (1993). Rotifers as predators on small ciliates. *Hydrobiologia* 255 247–253. 10.1007/BF00025845

[B27] Gutiérrez-Manero F. J. Ramos-Solano B. Probanza A. Mehouachi J. R Tadeo F. Talon M. (2001). The plant-growth-promoting rhizobacteria *Bacillus pumilus* and *Bacillus licheniformis* produce high amounts of physiologically active gibberellins. *Physiol. Plant.* 111 206–211. 10.1034/j.1399-3054.2001.1110211.x

[B28] Hamada M. Matar A. Bashir A. (2015). Carbaryl degradation by bacterial isolates from a soil ecosystem of the gaza strip. *Braz. J. Microbiol.* 46 1087–1091. 10.1590/S1517-838246420150177 26691466 PMC4704642

[B29] Hazem A. Manar A. (2003). Genetic polymorphism by RAPD-PCR and phenotypic characteristics of isolated thermotolerant Bacillus strains from hot spring sources. *New Microbiol.* 26 249–256. 12901420

[B30] Huber R. Eder W. Heldwein S. Wanner G. Huber H. Rachel R. (1998). *Thermocrinis ruber* gen. nov., sp. nov., a pink filament-forming hyperthermophilic bacterium isolated from Yellowstone National Park. *Appl. Environ. Microbiol.* 164 3576–3583. 9758770 10.1128/aem.64.10.3576-3583.1998 PMC106467

[B31] Jadhav S. S. David M. (2016). Biodegradation of flubendiamide by a newly isolated *Chryseobacterium* sp. strain SSJ1. *3 Biotech* 6:31. 10.1007/s13205-015-0347-9 28330102 PMC4713399

[B32] Kayasth M. Kumar V. Gera R. (2013). Exploring the potential of PGPR strain bacillus licheniformis to be developed as multifunctional biofertilizer. *Cent. Eur. J. Biol.* 2 12–17.

[B33] Kecha M. Benallaoua S. Touzel J. P. Bonaly R. Duchiron F. (2007). Biochemical and phylogenetic characterization of novel terrestrial hyperthermophilic archaeon pertaining to the genus *Pyrococcus* from an Algerian hydrothermal hot spring. *Extremophiles* 11 65–73. 10.1007/s00792-006-0010-9 16969710

[B34] Khan M. Patel C. B. (2007). Plant growth promoting the effect of *Bacillus firmus* strain NARS1 isolated from the central Himalayan region of India on *Cicer arietinum* at low temperature. *Egypt Afr. Crop Sci. Soc.* 8 1179–1181.

[B35] Kloepper J. W. Rodríguez-Kábana R. Mcinroy J. A. Young R. W. (1992). Rhizosphere bacteria antagonistic to soybean cyst (*Heterodera glycines*) and root-knot (*Meloidogyne incognita*) nematodes: identification by fatty acid analysis and frequency of biological control activity. *Plant Soil* 139 75–84. 10.1007/BF00012844

[B36] Kuddus M. Ramtekke P. W. (2012). Recent developments in production and biotechnological applications of cold-active microbial proteases. *Crit. Rev. Microbiol.* 38 380–388. 10.3109/1040841X.2012.678477 22849713

[B37] Kumar P. Patel S. K. Lee J. K. Kalia V. C. (2013). Extending the limits of Bacillus for novel biotechnological applications. *Biotechnol. Adv.* 31 1543–1561. 10.1016/j.biotechadv.2013.08.007 23954286

[B38] Lele O. H. Deshmukh P. V. (2016). Isolation and characterization of thermophilic *Bacillus* sp. with extracellular enzymatic activities from hot spring of Ganeshpuri, Maharashtra, India. *Int. J. Appl. Res.* 2 427–430.

[B39] Lorck H. (1948). Production of hydrocyanic acid by bacteria. *Physiol. Plant.* 1 142–146. 10.1111/j.1399-3054.1948.tb07118.x

[B40] Mallik M. A. B. Tesfai K. (1983). Compatibility of *Rhizobium japonicum* with commercial pesticides *in vitro*. *Bull. Environ. Contam. Toxicol.* 31 432–437. 10.1007/BF01622274 6640140

[B41] Martensson A. M. (1992). Effects of agrochemicals and heavy metals on fast-growing rhizobia and their symbiosis with small-seeded legumes. *Soil Biol. Biochem.* 24 435–445. 10.1016/0038-0717(92)90206-D

[B42] Miller C. S. Handley K. M. Wrighton K. C. Frischkorn K. R. Thomas B. C. Banfield J. F. (2013). Short-read assembly of full-length 16S amplicons reveals bacterial diversity in subsurface sediments. *PLoS One* 8:56018. 10.1371/journal.pone.0056018 23405248 PMC3566076

[B43] Mohammad B. T. Al Daghistani H. I. Jaouani A. Abdel-Latif S. Kennes C. (2017). Isolation and characterization of thermophilic bacteria from Jordanian hot springs: *Bacillus licheniformis* and *Thermomonas hydrothermalis* isolates as potential producers of thermostable enzymes. *Int. J. Microbiol.* 2017:6943952. 10.1155/2017/6943952 29163641 PMC5661075

[B44] Nagaraju J. Reddy K. D. Nagaraja G. M. Sethuraman B. N. (2001). Comparison of multilocus RFLPs and PCR-based marker systems for genetic analysis of the silkworm, *Bombyx mori*. *Heredity* 86 588–597. 10.1046/j.1365-2540.2001.00861.x 11554975

[B45] Nazina T. N. Lebedeva E. V. Poltaraus A. B. Tourova T. P. Grigoryan A. A. Sokolova D. S. H. (2004). *Geobacillus gargensis* sp. Nov., a novel thermophile from a hot spring, and the reclassification of *Bacillus vulcanic* as *Geobacillus vulcani* comb. nov. *Int. J. Syst. Evol. Microbiol.* 54 2019–2024. 10.1099/ijs.0.02932-0 15545427

[B46] Nei M. Stephens J. C. Saitou N. (1985). Methods for computing the standard errors of branching points in an evolutionary tree and their application to molecular data from humans and apes. *Mol. Biol. Evol.* 2 66–85. 2897060 10.1093/oxfordjournals.molbev.a040333

[B47] Panda A. K. Bisht S. S. Kumar N. S. Mandal S. D. (2015). Investigations on the microbial diversity of Jakrem hot spring, Meghalaya, India using cultivation-independent approach. *Genomics* 4 156–157. 10.1016/j.gdata.2015.04.016 26484205 PMC4535621

[B48] Perez C. Munoz-Garay C. Portugal L. C. Sanchez J. Gill S. S. Soberon M. (2007). *Bacillus thuringiensis* ssp. Israelis Cyt1Aa enhances an activity of Cry11Aa toxin by facilitating the formation of a pre-pore oligomeric structure. *Cell Microbiol.* 9 2931–2937. 10.1111/j.1462-5822.2007.01007.x 17672866 PMC3700374

[B49] Pikovskaya R. I. (1948). Mobilization of phosphorus in soil in connection with the vital activity of some microbial species. *Microbiology* 17 362–370.

[B50] Powell W. Morgante M. Andre C. Hanafey M. Vogel J. Tingey S. (1996). The comparison of RFLP, RAPD, AFLP and SSR (microsatellite) markers for germplasm analysis. *Mol. Breed.* 2 225–238. 10.1007/BF00564200

[B51] Rayar J. K. Arif M. Singh U. S. (2015). Relative efficiency of RAPD and ISSR markers in assessment of DNA polymorphism and genetic diversity among *Pseudomonas* strains. *Afr. J. Biotechnol.* 14 1097–1106. 10.5897/AJB10.1951

[B52] Reysenbach A. L. Ehringer M. Hershberger K. (2000). Microbial diversity at 83°C in the calcite springs, Yellowstone national park: another environment where the *Aquificales* and “Korarchaeota” coexist. *Extremophiles* 4 61–67. 10741838 10.1007/s007920050008

[B53] Reysenbach A. L. Wickham G. S. Pace N. R. (1994). Phylogenetic analysis of the hyperthermophilic pink filament community in Octopus spring, Yellowstone national park. *Appl. Environ. Microbiol.* 60 2113–2119. 7518219 10.1128/aem.60.6.2113-2119.1994 PMC201609

[B54] Roldàn-Ruiz I. Dendauw J. Van Bockstaele E. Depicker A. De Loose M. (2000). AFLP markers reveal high polymorphic rates in ryegrasses (*Lolium* spp.). *Mol. Breed.* 6 125–134. 10.1023/A:1009680614564

[B55] Rudresh D. L. Shivaprakash M. K. Prasad R. D. (2005). Effect of combined application of Rhizobium, phosphate solubilizing bacterium and *Trichoderma* spp. on growth, nutrient uptake and yield of chickpea (*Cicer aritenium* L.). *Appl. Soil Ecol.* 28 139–146. 10.1016/j.apsoil.2004.07.005

[B56] Sadfi N. Cherif M. Fliss I. Boudabbous A. Antoun H. (2001). Evaluation of bacterial isolates from salty soils and bacillus thuringiensis strains for the biocontrol of fusarium dry rot of potato tubers. *J. Plant Pathol.* 101–117. 10.4454/jpp.v83i2.1118

[B57] Saharan B. S. Verma S. (2014). Potential plant growth promoting activity of *Bacillus licheniformis* UHI(II)7. *Int. J. Microbial Res. Technol.* 2 22–27.

[B58] Sambrook J. Russel D. W. (2001). *Molecular Cloning: A Laboratory Manual*, 3rd Edn. Cold Spring Harbor, NY: Coldspring-Harbour Laboratory Press.

[B59] Satyanarayana T. Raghukumar C. Shivaji S. (2005). Extremophilic microbes: diversity and perspectives. *Curr. Sci.* 89 78–90.

[B60] Schaffer C. Franck W. L. Scheberl A. Kosma P. McDermott T. R. Messner P. (2004). Classification of isolates from locations in Austria and Yellowstone National Park as *Geobacillus tepidamans* sp. nov. *Int. J. Syst. Evol. Microbiol.* 54 2361–2368. 10.1099/ijs.0.63227-0 15545484

[B61] Schippers B. Bakker A. W. Bakker P. A. H. M. Van Peer R. (1990). Beneficial and deleterious effects of HCN-producing pseudomonads on rhizosphere interactions. *Plant Soil* 129 75–83. 10.1007/BF00011693

[B62] Sharma A. Pandey A. Shouche Y. S. Kumar B. Kulkarni G. (2009). Characterization and identification of *Geobacillus* spp. isolated from Soldhar hot spring site of Garhwal Himalaya, India. *J. Basic Microbiol.* 49 187–194. 10.1002/jobm.200800194 19025872

[B63] Siangbood H. Ramanujam P. (2011). A report on thermophilic *Cyanophyta* (Cyanobacteria) from Jakremhotspring, Meghalaya. *Int. J. Algae* 13 178–185. 10.1615/InterJAlgae.v13.i2.70

[B64] Soltanpour P. N. Workman S. M. (1981). Soil-testing methods used at Colorado State University Soil-Testing Laboratory for the Evaluation of Fertility, Salinity, Sodicity, and Trace-Element Toxicity. Fort Collins, CO: Colorado Technology Bulletin, 14–22.

[B65] Sridevi M. Veera Mallaiah K. (2007). Bioproduction of indole acetic acid by *Rhizobium* strains isolated from root nodules of green manure crop, *Sesbania sesban* (L.) Merr. *Iran. J. Biotechnol.* 5 178–182.

[B66] Srinivas T. N. R. Rao S. N. Reddy P. V. V. Pratibha M. S. Sailaja B. Kavya B. (2009). Bacterial diversity and bio prospecting for cold-active lipases, amylases and proteases, from culturable bacteria of Kongsfjorden and Ny-Ålesund, Svalbard, Arctic. *Curr. Microbiol.* 59 537–554. 10.1007/s00284-009-9473-0 19680721

[B67] Swain M. R. Naskar S. K. Ray R. C. (2007). Indole-3-acetic acid production and effect on sprouting of yam (*Dioscorea rotundata* L.) minisetts by *Bacillus subtilis* isolated from culturable cow dung microflora. *Pol. J. Microbiol.* 56 103–110. 17650680

[B68] Tamura K. Dudley J. Nei M. Kumar S. (2007). MEGA4: molecular evolutionary genetics analysis (MEGA) software version 4.0. *Mol. Biol. Evol.* 24 1596–1599. 10.1093/molbev/msm092 17488738

[B69] Tringe S. G. Hugenholtz P. (2008). A renaissance for the pioneering 16S rRNA gene. *Curr. Opin. Microbiol.* 11 442–446. 10.1016/j.mib.2008.09.011 18817891

[B70] Valverde A. Burgos A. Fiscella T. Rivas R. Velaz-quez E. Rodriguez-Barrueco C. (2006). Differential effects of inoculations with *Pseudomonas jessenii* PS06 (a phosphate-solubilizing bacteria) and *Mesorhizobium ciceri* C-2/2 strains on the growth and seed yield of chickpea under greenhouse and field conditions. *Plant Soil* 287 43–50. 10.1007/s11104-006-9057-8

[B71] Van den Burg B. B. (2003). Extremophiles as a source of novel enzymes. *Curr. Opin. Microbiol.* 6 213–218. 10.1016/S1369-5274(03)00060-2 12831896

[B72] Verma J. P. Tiwari K. N. Yadav J. Mishra A. K. (2016). Development of microbial consortia for growth attributes and protein content in micropropagated *Bacopa monnieri* (L.). *Proc. Natl. Acad. Sci. India Sec. B Biol. Sci.* 88 143–151.

[B73] Verma J. P. Yadav J. (2018). Implication of microbial consortium on biomass and yield of chickpea under sustainable agriculture. *Environ. Eng. Manage. J.* 17 513–522.

[B74] Verma J. P. Yadav J. Tiwari K. N. Jaiswal D. K. (2014). Evaluation of plant growth promoting activities of microbial strains and their effect on growth and yield of chickpea (*Cicer arietinum* L.) in India. *Soil Biol. Biochem.* 70 33–37. 10.1016/j.soilbio.2013.12.001

[B75] Verma J. P. Yadav J. Tiwari K. N. Kumar A. (2013). Effect of indigenous *Mesorhizobium* spp. and plant growth promoting rhizobacteria on yields and nutrients uptake of chickpea (*Cicer arietinum* L.) under sustainable agriculture. *Ecol. Eng.* 51 282–286. 10.1016/j.ecoleng.2012.12.022

[B76] Verma J. P. Yadav J. Tiwari K. N. Lavakush Singh V. (2010). The impact of Plant growth promoting rhizobacteria on crop production. *Int. J. Agric. Res.* 954–983. 10.3923/ijar.2010.954.983

[B77] Watanabe F. S. Olsen S. R. (1965). Test of an ascorbic acid method for determining phosphorus in water and NaHCO_3_ extracts from soil. *Soil Sci. Soc. Am. Proc.* 29 677–678. 10.2136/sssaj1965.03615995002900060025x

[B78] Yadav A. N. Verma P. Kumar M. Pal K. K. Dey R. Gupta A. (2015). Diversity and phylogenetic profiling of niche-specific Bacilli from extreme environments of India. *Ann. Microbiol.* 65 611–629. 10.1007/s13213-014-0897-9

[B79] Yadav S. Kaushik R. Saxena A. K. Arora D. K. (2011). Diversity and phylogeny of plant growth-promoting bacilli from the moderately acidic soil. *J. Basic Microbiol.* 51 98–106. 10.1002/jobm.201000098 21077114

[B80] Zhang D. Du G. Chen J. (2010). Fermentation production of microbial catalase and its application in textile industry. *Sheng Wu Gong Cheng Xue Bao* 26 1473–1481. 21284207

